# Dietary supplementation with *n-3* fatty acids from weaning limits brain biochemistry and behavioural changes elicited by prenatal exposure to maternal inflammation in the mouse model

**DOI:** 10.1038/tp.2015.126

**Published:** 2015-09-22

**Authors:** Q Li, Y O Leung, I Zhou, L C Ho, W Kong, P Basil, R Wei, S Lam, X Zhang, A C K Law, S E Chua, P C Sham, E X Wu, G M McAlonan

**Affiliations:** 1 Department of Psychiatry, The University of Hong Kong, Hong Kong, China; 2 State Key Laboratory for Cognitive and Brain Sciences, The University of Hong Kong, Hong Kong, China; 3 HKU-SIRI, The University of Hong Kong, Hong Kong, China; 4 Laboratory of Biomedical Imaging and Signal Processing, The University of Hong Kong, Hong Kong, China; 5 Department of Electrical and Electronic Engineering, The University of Hong Kong, Hong Kong, China; 6 Division of Diabetes, Endocrinology and Metabolism, Department of Medicine, Baylor College of Medicine, Houston, TX, USA; 7 Institute of Basic Medicine, Shandong Academy of Medical Sciences, Jinan, China; 8 Department of Neurology, Tongji Medical College, Huazhong University of Science and Technology, Wuhan, China; 9 Centre for Reproduction, Development and Growth, The University of Hong Kong, Hong Kong, China; 10 Genome Research Centre, The University of Hong Kong, Hong Kong, China; 11 Department of Forensic and Neurodevelopmental Sciences, Institute of Psychiatry, Psychology and Neuroscience, King's College, London, UK

## Abstract

Prenatal exposure to maternal immune activation (MIA) increases the risk of schizophrenia and autism in the offspring. The MIA rodent model provides a valuable tool to directly test the postnatal consequences of exposure to an early inflammatory insult; and examine novel preventative strategies. Here we tested the hypotheses that behavioural differences in the MIA mouse model are accompanied by *in vivo* and *ex vivo* alterations in brain biochemistry; and that these can be prevented by a post-weaning diet enriched with *n-3* polyunsaturated fatty acid (PUFA). The viral analogue PolyI:C (POL) or saline (SAL) was administered to pregnant mice on gestation day 9. Half the resulting male offspring (POL=21; SAL=17) were weaned onto a conventional lab diet (*n-6* PUFA); half were weaned onto *n-3* PUFA-enriched diet. *In vivo* magnetic resonance spectroscopy measures were acquired prior to behavioural tests; glutamic acid decarboxylase 67 (GAD_67_) and tyrosine hydroxylase protein levels were measured *ex vivo*. The main findings were: (i) Adult MIA-exposed mice fed a standard diet had greater N-acetylaspartate/creatine (Cr) and lower myo-inositol/Cr levels in the cingulate cortex *in vivo*. (ii) The extent of these metabolite differences was correlated with impairment in prepulse inhibition. (iii) MIA-exposed mice on the control diet also had higher levels of anxiety and altered levels of GAD_67_ *ex vivo*. (iv) An *n-3* PUFA diet prevented all the *in vivo* and *ex vivo* effects of MIA observed. Thus, *n-3* PUFA dietary enrichment from early life may offer a relatively safe and non-toxic approach to limit the otherwise persistent behavioural and biochemical consequences of prenatal exposure to inflammation. This result may have translational importance.

## Introduction

Schizophrenia and autism spectrum conditions are highly heritable, but environmental factors, such as exposure to maternal immune activation (MIA) in prenatal life, are thought to increase risk.^1, 2, 3, 4^ This epidemiological evidence has lead to the development of animal models and we, and others, have reported that MIA triggered by the viral analogue PolyI:C (POL) precipitates a brain and behavioural phenotype in rodent offspring which mirrors that observed in schizophrenia and related neurodevelopmental conditions such as autism.^5, 6, 7, 8, 9, 10^

Although the MIA model is a well-established experimental manipulation, behaviour testing is generally the only *in vivo* measure acquired and the underlying biochemical alterations in this model are inferred largely from *ex vivo* study. Proton magnetic resonance spectroscopy (^1^H MRS), however, permits the potential relationship between brain metabolites and behaviour to be studied in the same living animal. Although MRS studies in patient populations have yielded much information about brain chemistry in the living brain, it is not known whether prenatal inflammation is a risk factor for such changes. Therefore, we first wished to establish whether indeed *in vivo* changes in brain metabolites similar to those reported in the clinical condition are caused by prenatal inflammation; and then asked if these are correlated with behavioural differences.

Second, the MIA model provides a practical means to investigate adult outcomes of early life interventions, as rodents have a much shorter life span than people. Therefore, in this study we also took the opportunity to test the hypothesis that early dietary supplementation with *n-3* polyunsaturated fatty acid (PUFA) from weaning would prevent emergence of adult biochemical and behavioural differences triggered by MIA. We selected *n-3* PUFA because these fats are essential for the development of the central nervous system^11^ and they have robust anti-inflammatory properties.^12^ In particular, *n-3* PUFA inhibits production of interleukin-6,^12^ which is a key driver of pathology in the MIA model.^13^ Finally, *n-3* PUFA shows some initial promise in the clinical setting—it reduces symptoms in young people with ‘subthreshold' schizophrenia^14^—but direct experimental evidence is needed.

Therefore, we used *in vivo* MRS to quantify anterior cingulate cortex biochemistry in adult mice exposed to either MIA or saline (SAL) in prenatal life. *A priori*, we predicted that MIA exposure would alter levels of N-acetylaspartate (NAA) and myo-inositol (mIns), as differences in these neuronal and astrocytic markers, respectively, have been linked to neurodevelopmental disorders. We also examined the relationship between these metabolites and behaviour in the same animals. Finally we measured *ex vivo* levels of the GABA marker, glutamic acid decarboxylase 67 (GAD_67_) and the dopamine marker, tyrosine hydroxylase (TH), as both have been reported to be altered in neurodevelopmental conditions.^15, 16, 17, 18, 19, 20, 21^ Half the animals in each group (MIA or SAL) received an *n-3* PUFA diet post weaning. We predicted that an *n-3* PUFA dietary intervention would limit the behavioural and biochemical consequences of prenatal MIA.

## Materials and methods

Female and male C57BL6/N mice were bred and mated by The University of Hong Kong, Laboratory Animal Unit. Timed-pregnant mice were held in a normal light–dark cycle (light on at 0700 hours), and temperature and humidity-controlled animal vivarium. All animal procedures were approved by the Committee on the Use of Live Animals in Teaching and Research (CULATR) at The University of Hong Kong.

**Figure 1:**
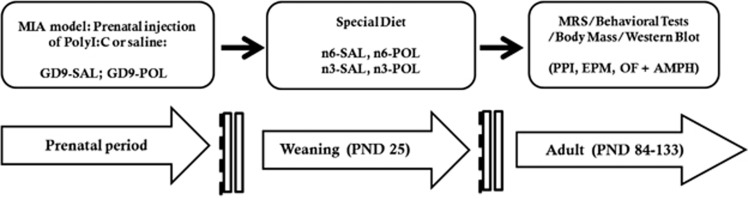
Experimental design. AMPH, amphetamine; EPM, elevated plus maze; GD, gestation day; MIA, maternal immune activation; OF, open field; PND, postnatal day; POL, PolyI:C; PPI, prepulse inhibition; PUFA, polyunsaturated fatty acids; SAL, saline. Groups: n3-POL, prenatal PolyI:C-exposed offspring treated with *n-3* PUFA; n6-POL, prenatal PolyI:C-exposed offspring treated with *n-6* PUFA; n3-SAL, prenatal saline-exposed offspring treated with *n-3* PUFA; n6-SAL, prenatal saline-exposed offspring treated with n6-polyunsaturated fatty acids (*n-6* PUFA) control diet.

The MIA model was generated following procedures previously reported.^7, 10^ The estimation of sample size for this study was based on the data from exploratory studies of behaviour. Allowing for randomized block analysis of variance power analysis with alpha=0.05 and power=0.80 using PASS software (NCSS, Kaysville, UT, USA) indicated that eight mice should be assigned to each group. A dose of 5 mg kg^−1^ POL in an injection volume 5 ml kg^−1^, prepared on the day of injection was administered to pregnant mice on gestation day 9 via the tail vein under mild physical constraint. Control animals received an injection of 5 ml kg^−1^ 0.9% SAL. The resulting male offspring (POL *n*=21; SAL *n*=17) from six litters were weaned on postnatal day 25, then randomly divided into two groups. Half were fed on diets enriched with *n-3* PUFAs and half were fed a standard (control) lab diet until the end of the study. (See details in Table 1 and Figure 1). The latter ‘*n-6* PUFA' control diet had the same calorific value and total fat content as *the n-3* PUFA diet. The diets were custom prepared and supplied by Harlan Laboratories (Madison, WI, USA). The *n-6* and *n-3* PUFAs were derived from corn oil or menhaden fish oil, respectively. The *n-6* PUFA control diet, was based on the standard AIN-93G rodent laboratory diet,^22^ and contained 65 g kg^−1^ corn oil and 5 g kg^−1^ fish oil with an approximate (n6)/(n3) ratio of 13:1. The *n-3* PUFA diet contained 35 g kg^−1^ corn oil and 35 g kg^−1^ fish oil with an approximate (n6)/(n3) ratio of 1:1.^23^

### ^1^H-MRS acquisition

**Figure 2:**
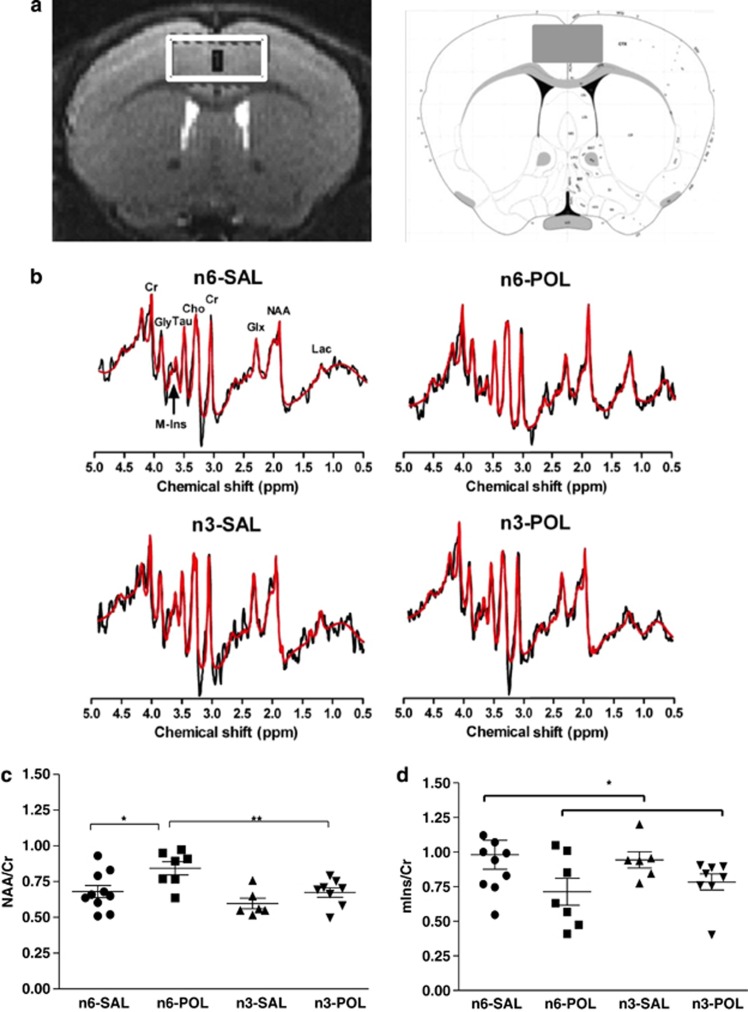
Representative MRS voxel-of-interest (VOI) and MRS metabolite levels in adult offspring exposed to prenatal inflammation or saline, with or without *n-3* PUFA intervention in peri-adolescence. Representative MRS voxel-of-interest (VOI) and spectra acquired in adult offspring: (**a**) Voxel-of-interest with the size of 1.2 × 2.6 × 2.5 mm^3^ was positioned in the anterior cingulate of each mouse. Mouse atlas reference of VOI is from Allen Institute:^25^ (**b**) Representative *in vivo* ^1^H spectra with quantum estimation (QUEST) fitting from the anterior cingulate in each group. MRS raw spectrum is shown in black; estimate fitting is shown in red. MRS metabolite levels: (**c**) Greater N-acetylaspartate (NAA)/creatine (Cr) in *n-6* PolyI:C-exposured group compared with all other groups (*P<0.05, **P⩽0.01); *n-3* PUFA prevents the rise in NAA/Cr caused by prenatal PolyI:C exposure. (**d**) Lower myo-inositol (mIns)/Cr in PolyI:C-exposed group compared with Saline group. Groups: n3-POL, prenatal PolyI:C-exposed offspring treated with *n-3* PUFA; n6-POL, prenatal PolyI:C-exposed offspring treated with *n-6* PUFA; n3-SAL, prenatal saline-exposed offspring treated with *n-3* PUFA; n6-SAL, prenatal saline-exposed offspring treated with n6-polyunsaturated fatty acids (*n-6* PUFA) control diet. MRS, magnetic resonance spectroscopy; POL, PolyI:C; PUFA, polyunsaturated fatty acids; SAL, saline.

The MRS procedure followed that described in detail in our previous report.^24^ Twelve-week-old mice were scanned using a 7 T scanner with a maximum gradient of 360 mT m^−1^ (70/16 PharmaScan, Bruker Biospin, Ettlingen, Germany) and a four channel mouse brain surface coil. Animals were anaesthetised during scanning with isoflurane/air mixture at 3% for induction and 1.5% for maintenance via a nose cone. Three T2-weighted scout images were first acquired with a rapid acquisition relaxation enhanced sequence (repetition time/echo time=4200/36 ms, rapid acquisition relaxation enhanced factor=8, spatial resolution=0.109 × 0.109 × 0.48 mm^3^) for the localisation of the voxel-of-interest. A 1.2 × 2.6 × 2.5-mm^3^ voxel-of-interest was placed over the cingulate cortex (Figure 2a). The voxel used was necessarily larger than the actual mouse anterior cingulate cortex and included part of the motor cortex, but the majority of the scanned volume was cingulate cortex.^24^ After first- and second-order localised shimming with a FieldMap-based procedure, a full-width half-maximum linewidth of water signal of ⩽15 Hz was achieved. The water signal was suppressed by VAPOR (variable RF pulses with optimised relaxation delays). A point-resolved spectroscopy sequence combined with outer volume suppression was used for spectrum acquisition using repetition time/echo time=2500/14 ms, spectral bandwidth=4 kHz, 2048 data points and 256 averages. Research staff involved in MRS scan and data collection were ‘blinded' to the group assignment.

### ^1^H-MRS spectral analysis

MR spectra were processed using the jMRUI software (http://www.mrui.uab.es/mrui/). The raw data were apodized with a 15-Hz Gaussian filter and phase corrected. The residual water signal was filtered out using the Hackel–Lanczos singular value decomposition (HLSVD) algorithm. Chemical shifts of peaks were assigned with reference to the CH3-group of NAA at 2.02 p.p.m. Metabolite area under the peak was quantified by quantum estimation method with subtraction approach for background modelling. The metabolite parameters were decorrelated from the background with truncation of initial data points, given that macromolecules and lipids signals decay rapidly across the time-domain. The numerical time-domain model functions of 11 metabolites, including choline (Cho), creatine (Cr), glutamate (Glu)+glutamine=Glx, glycine (Gly), lactate (Lac), mIns, combined NAA Peak (NAA+N-acetylaspartylglutamate: NAAG) and taurine (Tau), were used as prior knowledge in quantum estimation (Figure 2b). These metabolite model signals were quantum mechanically simulated in nuclear magnetic resonance spectra calculation using operators (NMR-SCOPE). Errors in measurement of noise and inadequate modelling of the overlapping background signal were calculated by the Cramér–Rao lower bounds, which were used to assess the reliability of metabolite quantitation. The quantification was considered appropriate only when the corresponding bound was below 25%.^24, 26, 27^ Total Cr (creatine and phosphocreatine) was used as the internal spectral reference. There is a narrow spectral gap between NAA and NAAG (2.04 p.p.m.), therefore the combined signals of NAA and NAAG are conventionally interpreted as representing NAA in both preclinical and human studies.^24, 28^ Group differences in NAA/Cr, Cho/Cr, Glx/Cr, mIns/Cr, Gly/Cr Lac/Cr and Tau/Cr ratios were analysed.

### Behavioural tests

#### PPI of the acoustic startle response

The procedures and testing parameters for evaluation of prepulse inhibition (PPI) have been fully described previously.^7^ In brief, the PPI paradigm was conducted using startle chambers for mice (San Diego Instruments, San Diego, CA, USA). In a test session, a mix of pulse-alone (100, 110 and 120 dBA), prepulse-plus-pulse (3 prepulse options × 3 pulse options), prepulse-alone (71, 77 and 83 dBA) and no-stimulus (background noise, 65 dBA) trials were presented. PPI was calculated by the following formula: 100% × [1-(mean reactivity on prepulse-plus-pulse trials/mean reactivity on pulse-alone trials)] and the mean %PPI across all three prepulse and three pulse conditions was examined. Thereafter, group contrasts in individual prepulse/pulse conditions were explored if appropriate.

#### Elevated plus maze

The elevated plus maze test is based on the aversion of mice to open and high spaces, and is used for measurement of anxiety.^29^ The plus maze consisted of four 30-cm-long arms radiating out from a central square measuring 5 × 5 cm. Two ‘closed' arms were enclosed by 14-cm-high opaque walls from all sides except the side adjoining the central square. The other two ‘open' arms were exposed, and the outer rim of each arm was guarded by a perimeter border of 1 mm. The maze was located in a dimly lit behaviour test room and was elevated at a height of 70 cm above floor level.^30^ The mice were gently placed in the centre of the maze facing one of the open arms. It was allowed to move freely for 10 min. The dependent measures were: (a) duration of time spent in the open arms, (b) frequency of open arm entries, and (c) duration of time spent, and frequency of entries into, the open arms as a percentage of the total duration and total arm entries. A digital camera was mounted above the maze and images were analysed using Ethovision tracking system (VersionXT 7.1, Noldus, Wageningen, The Netherlands).

#### Locomotor response to amphetamine

The apparatus comprised four identical cubes made of Plexiglas with a white opaque bottom, each measuring 40 × 40 × 40 cm. In the middle of the floor, a central arena (13.5 cm × 13.5 cm) was demarcated by a red line.^31^ Mice received 0.9% NaCl i.p., to control for injection stress, and were returned to the open field for 30 min. Afterwards, they were carefully removed, given an i.p. injection of amphetamine, and returned to the open field for a further 90 min. Amphetamine sulphate (Sigma, St Louis, MO, USA), was dissolved in 0.9% NaCl solution on the day of testing to obtain 2.5 mg kg^−1^, in a volume of 5 ml kg^−1^. Locomotor activity was recorded after SAL and amphetamine injection using Ethovision tracking system. The dependent measures were the total distance travelled during four time blocks: SAL (30 min), amphetamine (Amph)-block 1 (30 min), Amph-block2 (30 min) and Amph-block3 (30 min).

### Body mass

Measurements of body weight, lean tissue, body fat and body fluid were acquired after behavioural tests using the minispec LF90 (Bruker optics, Billerica, MA, USA), an NMR analyser for whole body composition assay of live, unanesthetized mice.

### Western blot

Medial prefrontal cortex (mPFC), caudate putamen (CP) and nucleus accumbens (NAc) were harvested from mice 1 week after amphetamine challenge to minimise residual pharmacological effects on brain. Brain slices were obtained using a mouse brain matrix, and tissue was taken using fine forceps. Samples were homogenised in RIPA buffer with protease inhibitor cocktail (Sigma, P 8340) and protein concentration was determined by Thermo protein assay (Thermo Scientific, Waltham, MA, USA, 22660). All the samples were equalized to 20 μg. Procedures for western blot followed those described previously.^32, 33^ The primary antibodies to the following proteins: TH (1:1000, sc-14007, Santa Cruz, Dallas, TX, USA), GAD67 (1:1000, ab52249, Abcam, Cambridge, UK), and β-actin (horseradish peroxidase) (1:30 000, ab49900, Abcam) were incubated with the membrane in the antibody dilution buffer with gentle agitation overnight at 4 °C, then incubated with the secondary antibody (1:2000 dilution, P044801, Dako, Glostrup, Denmark) for 1 h at room temperature. The signal was revealed by a chemiluminescent detection method (ECL, Amersham, Buckinghamshire, UK). The intensities of the bands were quantified using ImageJ (NIH, Bethesda, MD, USA).^33^ Research staff involved in experimental performance and analysis were ‘blinded' to the group assignment.

### Statistical analysis

MRS: A 2 × 2 [Prenatal treatment (SAL and POL) × Diet (*n-3* or *n-6* PUFA)] multivariate general linear model (GLM) using SPSS 20 was applied followed by *post hoc* *t*-tests. Metabolites that had a Cramér–Rao lower bound value >25% were excluded from analysis. Based on this criterium, one mouse from n6-POL group and two mice from n3-SAL group were excluded. Thus the final numbers for MRS analysis were: PolyI:C group *n*=15 (*n6*-POL=7; n3-POL=8); Saline group *n*=16 (*n6*-SAL=10; n3-SAL=6). Levene's test was applied to test for equality of variance. Results were considered to be significant at *P*<0.05.

Body mass and western blot were analysed using a 2 (prenatal treatment) × 2 (diet) GLM.

Behavioural video data analysis and data collection were done by research staff ‘blinded' to the group assignment of each animal. Behavioural data was analysed using either GLM for normal distributions and Kruskal–Wallis nonparametric one-way analysis of variance otherwise. Depending on the distribution of the data, *post hoc* analyses were performed using Mann-Whitney comparisons or Fisher's least significant difference *post hoc* comparisons wherever appropriate. Levene's test was applied to test for equality of variance wherever appropriate. Results were considered to be significant at *P*<0.05.

One-tailed partial linear correlation analyses controlling for groups were planned to evaluate predicted relationships between MRS metabolites and behavioural indices shown to have significant group differences (when normally distributed).

## Results

### Brain metabolites measured by ^1^H-MRS

There was a significant main effect of prenatal treatment on NAA/Cr (F(1, 27)=8.109, *β*=0.8132, *P*<0.01); and a significant main effect of diet on NAA/Cr (F(1, 27)=9.059, *β*=0.8548, *P*<0.01). *Post hoc* *t*-tests confirmed that NAA/Cr in n-6 POL group was significantly higher than the n6-SAL group (*P*<0.05); NAA/Cr levels in n3-POL were significantly lower than *n6*-POL (*P*=0.01) and not different from n3-SAL (*P*=0.151). Thus, *n-3* PUFA diet prevented a POL-induced elevation of NAA/Cr. See Figure 2c.

There was a significant effect of prenatal treatment on mIns/Cr (F(1, 27)=5.425, *β*=0.6355, *P*<0.05); MIA exposure lowered mIns/Cr and this was most prominent in *n6*-POL when compared with *n6*-SAL. However, this contrast did not reach statistical significance *post hoc* (*P*=0.096) and should therefore be treated with caution. There were no differences in n3-POL animals and n3-SAL, again suggesting *n-3* PUFA diet limited a lowering of mIns in MIA-exposed animals (Figure 2d).

There were no statistically significant differences in the concentrations of other MRS metabolites sampled (see Supplementary Table 1).

### Behavioural tests

#### PPI of the acoustic startle response

There were no significant differences in baseline pulse- or prepulse-elicited reactivity (Supplementary Figure 1).

**Figure 3:**
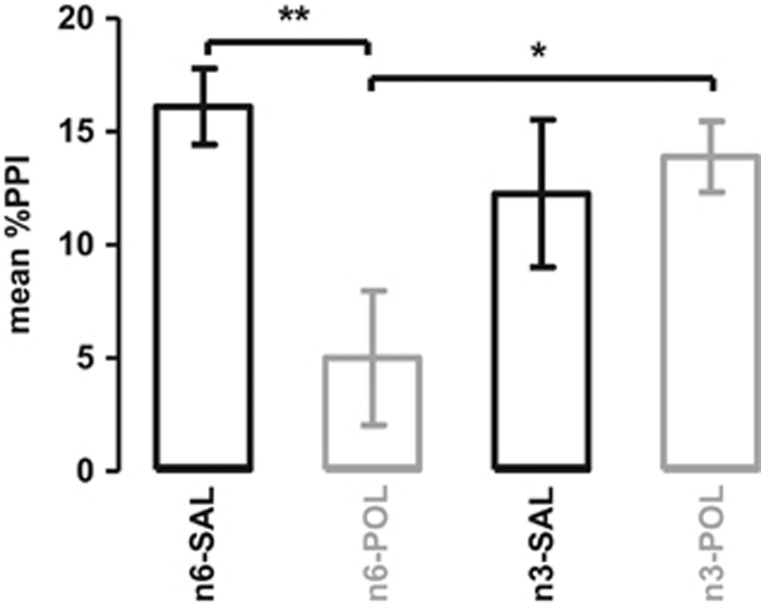
Mean percentage prepulse inhibition (%PPI). The bar plot represents mean %PPI across all prepulse and pulse stimuli. All values are mean±s.e.m. *P<0.05, **P<0.01. Groups: n3-SAL, prenatal saline-exposed offspring treated with *n-3* PUFA; n6-SAL, prenatal saline-exposed offspring treated with n6-polyunsaturated fatty acids (*n-6* PUFA) control diet.

The main effect of diet on mean %PPI approached significance (F(1, 34)=3.85, *P*=0.058) and there was a significant interaction between diet and prenatal treatment (F(1, 34)=6.95, *P*<0.05). *Post hoc* comparisons indicated that this was explained by significantly impaired PPI in the n6-POL group (*P*<0.01), but ‘improved' PPI in the n3-POL group (*P*<0.05) (Figure 3). There was no relationship between body weight and %PPI. Thus *n-3* PUFA diet prevented PPI impairment caused by MIA exposure.

#### Elevated plus maze

Elevated plus maze data in open arms expressed as medians and interquartile ranges is shown in Table 2. Kruskal–Wallis analysis of variance revealed a significant group (n6-SAL, n6-POL, n3-SAL, n3-POL) difference of percentage (%) time spent on open arms (*χ*^2^=8.264, df=3, *P*<0.05). The main effect of group on % of entries into open arms just failed to reach significance (*χ*^2^=7.418, df=3, *P*=0.06). *Post hoc* Mann-Whitney comparisons confirmed that, compared with n6-SAL, n6-POL mice spent less time in the open arms (*P*⩽0.05) (Table 2), which suggested MIA-exposed mice were ‘anxious'. However, there was no difference between n3-SAL and n3-POL groups on these measures, indicating that n3-PUFA diet attenuated ‘anxiety' in the MIA-exposed group.

#### Locomotor response to SAL and amphetamine

As expected, amphetamine increased locomotion in each group (n6-SAL, n6-POL, n3-SAL and n3-POL) (F(3, 102)=5.868, *P*<0.01), (See Supplementary Figure 2). *Post hoc* comparisons confirmed a maximal response to amphetamine after 30 min in all groups (*P*<0.05). However, there were no differences between groups in the response to amphetamine (Supplementary Figure 2).

### Body mass

POL-exposed animals were smaller than SAL-exposed controls (F(1, 34)=7.024, *P*<0.05). *n-3* PUFA diet increased weight and body mass (lean and fluid) in both groups, as shown by a main effect of diet on weight (F(1, 34)=6.725, *P*<0.05), lean body mass (F(1, 34)=12.587, *P*=0.001) and fluid mass (F(1, 34)=6.862, *P*<0.05). *Post hoc t*-test comparisons confirmed that n3-SAL mice gained more weight and body mass when compared with n6-SAL (weight *P*=0.001, lean *P*<0.001, fluid *P*<0.0001) or when compared with n3-POL (weight *P*<0.01, lean *P*<0.01, fluid *P*<0.01). (Table 3).

### Western blot quantification of GAD_67_

NAc: there was a significant prenatal treatment × diet interaction in GAD_67_ levels (F(1, 34)=11.763, *P*<0.01). *Post hoc* *t*-tests confirmed that this was due to lower GAD_67_ in n6-POL compared with n6-SAL (*P*<0.0001). Importantly, *n-3* PUFA diet significantly increased GAD_67_ in the n3-POL group compared with the n6-POL group (*P*<0.05). Thus, *n-3* PUFA diet effectively restored GAD_67_ to ‘control' levels, as there was no statistical difference between n3-POL and n3-SAL groups. However, diet lowered GAD_67_ in n3-SAL relative to n6-SAL (*P*<0.01).

The pattern of differences in GAD_67_ levels was similar in the CP, though these differences did not quite reach statistical significance: main effect of prenatal treatment (F(1, 34)=3.492, *P*=0.07); diet × prenatal treatment (F(1, 34)=3.556, *P*=0.068). *Post hoc* testing, however, confirmed a lower level of GAD_67_ in n6-POL compared with n6-SAL (*P*<0.01).

**Figure 4:**
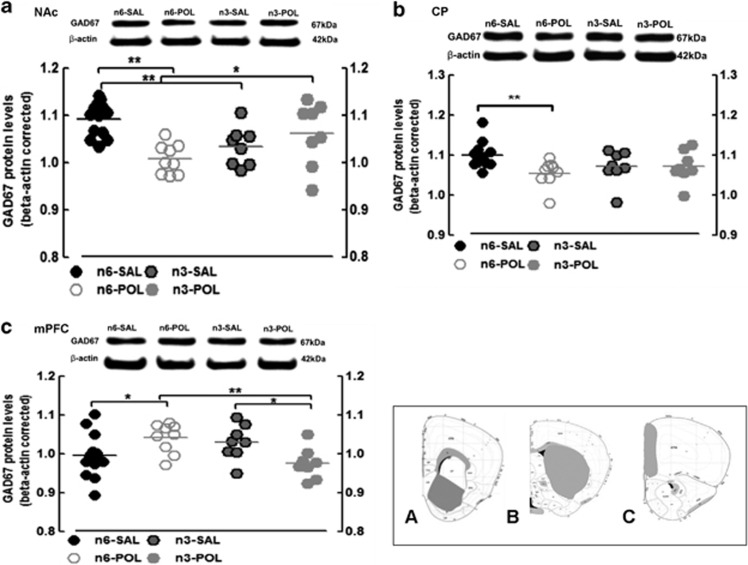
GAD67 protein levels. (**a**) GAD67 protein level in nucleus accumbens (NAc), (**b**) caudate putamen (CP) and (**c**) medial prefrontal cortex (mPFC). β-actin is shown as a control for comparison. All values are mean±s.e.m. *P<0.05, **P<0.01. Histology panels represent coronal mouse atlas reference from Allen Institute,^34^ indicating regions-of-interest dissected for analyses (A=NAc, B=CP, C=mPFC). Groups: n6-POL, prenatal PolyI:C-exposed offspring treated with *n-6* PUFA; n3-SAL, prenatal saline-exposed offspring treated with *n-3* PUFA; n6-SAL, prenatal saline-exposed offspring treated with n6-polyunsaturated fatty acids (*n-6* PUFA) control diet.

In the mPFC region, the pattern of findings was in the opposite direction. There was a significant diet × prenatal treatment interaction (F(1, 34)=10.518, *P*<0.01) which was explained by greater GAD_67_ in n6-POL compared with n6-SAL; n3-PUFA diet ‘reversed' this abnormal elevation in the n3-POL group compared with n6-POL (*P*<0.01) (Figure 4).

The level of TH in NAc, CP and mPFC was not altered by MIA exposure, nor by dietary intervention. (Supplementary Figure 3).

### Correlation analyses of *in vivo* measures

**Figure 5:**
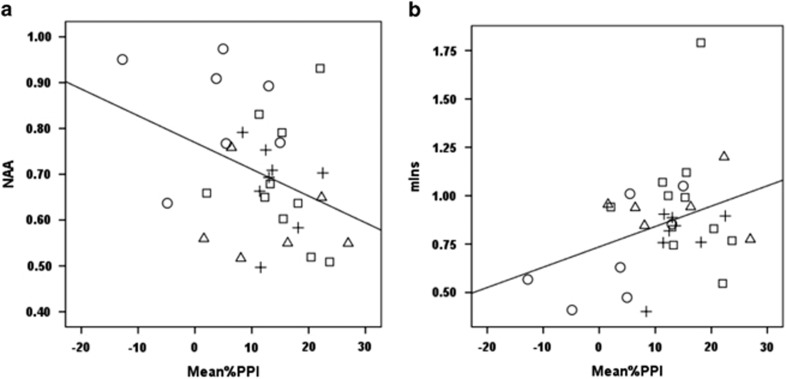
Metabolite and behaviour correlation. Mean %PPI and NAA/Cr correlation (**a**); Mean %PPI and mIns/Cr correlation (**b**). □,○,△,+ refer to n6-SAL, n6-POL, n3-SAL and n3-POL, respectively. Groups: n6-POL, prenatal PolyI:C-exposed offspring treated with *n-6* PUFA; n3-SAL, prenatal saline-exposed offspring treated with *n-3* PUFA; n6-SAL, prenatal saline-exposed offspring treated with n6-polyunsaturated fatty acids (*n-6* PUFA) control diet.

The level of NAA/Cr in the prefrontal cortex was significantly negatively correlated with PPI (*γ*=−0.365, df=28, *P*<0.05); that is, abnormal elevation of NAA in the prefrontal cortex was associated with greater PPI impairment (Figure 5a). In addition, the level of mIns/Cr in the prefrontal cortex was significantly positively correlated with PPI impairment (*γ*=0.355, df=28, *P*<0.05); thus abnormally low levels of mIns/Cr were associated with greater PPI impairment (Figure 5b).

## Discussion

We believe this study provides the first evidence that postnatal behavioural differences in offspring exposed to prenatal POL are accompanied by metabolite differences in the cingulate cortex; and that both the behavioural and metabolite sequelae can be limited by an *n-3* PUFA-enriched diet from adolescence. Specifically, NAA/Cr was higher and mIns/Cr was lower in adult mice exposed to prenatal POL challenge, and the extent of these differences was correlated with impairments in PPI. MIA-exposed mice were also more anxious in the elevated plus maze. These *in vivo* differences were accompanied by *ex vivo* differences in GAD_67_—an increase in the prefrontal cortex and a decrease in the striatum of mice exposed to MIA. However, an *n-3* PUFA diet from weaning attenuated both *in vivo* behavioural and metabolite abnormalities and *ex vivo* biochemical differences caused by MIA.

### *In vivo* MRS

NAA has been reported to be lower in the medial temporal regions, hippocampus and the frontal lobe in people with schizophrenia.^35, 36^ However, these studies have often included people in the chronic stages of illness and in receipt of medication. More recent analyses suggest that NAA may not be lower in first episode psychosis patients,^37^ or individuals at ultra-high risk of schizophrenia;^38^ and NAAG or NAA/Cr may even be higher in the ACC or dorsolateral prefrontal region of younger individuals with schizophrenia or ultra-high risk.^39, 40^ Thus, the picture in schizophrenia may depend on the stage of illness examined, as well as exposure to medication; NAA may be elevated early in the illness or in adolescents or young adulthood, but lowered in chronic stages. The advantage of the animal model is that, in our young adult mice we can confidently say that the metabolite differences observed are not due to disease ‘chronicity' or medication exposure.

What higher NAA means is not completely clear. The synthesis of NAA is exclusively carried out in mitochondria,^41^ but the deacetylation of NAA (by aspartoacylase) takes place in oligodendrocytes.^42^ Therefore, although NAA is often assumed to reflect neuronal health,^43, 44^ its levels may also be altered by oligodendrocyte abnormalities.^45^ For example, myelin degeneration due to aspartoacylase deficiency leads to an increase in NAA levels in the leukodystrophy, Canavan's Disease.^46^ Thus, NAA may reflect disruption of the neuronal-oligodendrocyte unit and, in line with this we and others have reported white matter structural and gene expression anomalies in the MIA model^10, 47^ that are broadly similar to those found in schizophrenia and related conditions.^48, 49, 50, 51, 52^

In addition to differences in NAA, we found lower mIns/Cr in POL-exposed animals on the control diet. Myo-inositol is a marker for astrocytes, and there is increasing evidence of astrocytic dysfunction in psychiatric disorders, such as depression,^53, 54^ schizophrenia^55, 56^ or bipolar disorder.^56^ Astrocytic loss/dysfunction in the prefrontal cortex of rats has been reported to impair cognitive function and this is thought to be consistent with a role for astrocytes in psychiatric disorders.^57^ In addition, myo-inositol is an important component of the phosphatidylinositol second messenger system (PI-cycle), and alterations in PI-cycle activity and oxidative stress^58^ have also been implicated in psychiatric disorders.^59^ The action of *n-3* PUFA diet as an antioxidant^60^ may limit this pathology^61, 62^ and may explain additional benefits of *n-3* PUFA diet beyond the neuronal-oligodendrocyte unit.

### Behaviour

PPI of the acoustic startle response is widely used to investigate sensorimotor gating and information processing across species.^63, 64^ This study confirmed the impact of prenatal immune activation on sensorimotor gating function^7, 31^ and anxiety-like behaviour,^65^ and these behavioural impairments were thought to mimic those reported in neurodevelopmental disorders.^63, 66, 67, 68, 69, 70^

Critically, we found that early administration of *n-3* PUFA from peri-adolescence, not only limited metabolite alterations in mice exposed to MIA, but also attenuated behavioural abnormalities in adulthood. This adds to the evidence that omega-3 improves sensorimotor gating function in a pharmacological and genetic rodent model of schizophrenia,^60,71^ and that long-term *n-3* PUFA administration can suppress anxiety-like behaviour.^72^

PPI impairment was most evident in animals with highest levels of NAA/Cr. NAA has been shown to cause oxidative damage following intracerebroventricular injection,^73^ and a disruption of oxidative metabolism has been implicated in PPI impairment.^74^ *n-3* PUFA diet may therefore prevent PPI deficit by ‘protecting' against on-going oxidative stress in the MIA model.

PPI impairment was also a feature of animals with lowest levels of mIns. This fits with evidence linking *Inositol monophosphatase 1* (*Impa1*) gene—a regulator of *myo-inositol* synthesis—to PPI;^75^ and a report that lithium, an inhibitor of *Impal*, alters measures of auditory gating.^76^ Taken together, *n-3* PUFA diet may prevent PPI deficit by additional effects on the metabolism of mIns.

However, in contrast to others,^31, 77^ we did not observe hypersensitivity to amphetamine in this MIA mice model. This may be a consequence of our choice of strain—the C57BL6/N mice used here have been reported to have lower baseline activity compared with the C57BL6/J mouse strain used in those other studies.^78^ In addition, we elected to use a relatively low dose amphetamine challenge (2.5 mg kg^−1^) to avoid potential confounds of stereotypy. We cannot exclude the possibility that a higher dose of amphetamine would have revealed greater separation between the groups.

### GAD_67_/TH

POL exposure lowered *ex vivo* GAD_67_ protein levels in the NAc and CP, consistent with postmortem findings in schizphrenia;^79^ and *n-3* PUFA supplementation prevented this. GABA inhibitory interneuron dysfunction is thought to arise from oxidative damage during development^80^ and certainly prenatal MIA represents one possible trigger. In contrast, *n-3* PUFA is known to protect neurons from oxidative stress,^27^ and this may contribute to its beneficial action here. The GABAergic abnormalities observed here likely contribute to the pattern of behavioural differences. For example, it is well-established that striatal GABAergic neurotransmission is involved in PPI ^81, 82^ and anxiety-like behaviour.^82^

Neither MIA or diet altered TH, a marker for dopamine synthesis. However, we emphasise that the western blot findings, particularly for TH, should be interpreted with caution. Clinical evidence shows altered dopaminergic abnormalities during the early stages of schizophrenia, which can be present even in ultra-high risk subjects.^83^ Besides, others have reported effects of MIA on dopaminergic system in drug-naive animals.^34, 84, 85, 86^ One possible explanation for our contradictory findings may be the exposure of our animals to a single administration of amphetamine. Although we ensured a 1 week ‘wash-out' period before death, a single administration of amphetamine (at identical or similar doses) can induce dopaminergic sensitization,^87, 88^ which may have masked the effects of MIA or diet on the dopamine system.

### Overall metabolism

MIA exposure led to smaller offspring, suggesting this prenatal insult had a systemic impact. Similar findings have been reported in POL-exposed rats.^89^ These are preliminary data but we suggest they should prompt more detailed studies of metabolic function in offspring exposed to MIA, as a wide range of metabolic abnormalities have been identified in patients with psychiatric conditions.^90^ In this study, *n-3* PUFA improved weight in MIA-exposed mice, but it also increased weight and body mass (lean and fluid) in the control mice. However, we cannot say whether the effect in control animals is a positive or negative influence on overall health.

### Limitations

We first acknowledge that the sample size of current study is modest. That said, the effect sizes observed were large and mulitimodal measures acquired from the same animals allowed exploration of relationships between behaviour and biochemistry, which were consistent with predictions. Second, we examined only adult male offspring in the current study. The decision to direct finite experimental resources to males was made because males with neurodevelopmental disorders such as autism outnumber females; and there is evidence that the male foetus is more vulnerable to environmental exposures such as inflammation in prenatal life.^91, 92^

## Conclusions

To the best of our knowledge, these experiments provide the first direct experimental evidence that *in vivo* metabolic changes and the behaviour effects of MIA are linked. They also support a beneficial effect of *n-3* PUFA diet from weaning in this animal model of neurodevelopmental disorders. We suggest that further study of the protective effects of *n-3* PUFA diet is warranted as it may open new avenues for prevention in neurodevelopmental psychiatric disorders.

## Figures and Tables

**Table 1:** Sample size of each experimental condition and the sequence of different experiments in male offspring

	*Experiments*	*n6-SAL*	*n6-POL*	*n3-SAL*	*n3-POL*	*Age (days)*
Neuroimaging	MRS	10	7	6	8	84–89
Behavioural tests (order of the tests)	Prepulse inhibition	13	9	8	8	98–105
	Elevated plus maze	13	9	8	8	112–119
	Open field test and amphetamine-induced locomotor activity	13	9	8	8	119–126
Physiological test	Body mass	13	9	8	8	133
Neurochemical test	Western blot	13	9	8	8	133

Abbreviation: MRS, magnetic resonance spectroscopy; n6-POL, prenatal PolyI:C-exposed offspring treated with *n-6* PUFA; n3-SAL, prenatal saline-exposed offspring treated with *n-3* PUFA; n6-SAL, prenatal saline-exposed offspring treated with n6-polyunsaturated fatty acids (*n-6* PUFA) control diet; PUFA, polyunsaturated fatty acids; Sal, saline.

**Table 2:** Summary of the offspring's performance in the EPM in adulthood in MIA mice with or without *n-3* PUFA interventions in peri-adolescence

*Groups*	*Time spent on open arms (s)*	*Open arm entries*	*% Time spent on open arms*	*% Entries of open arms*
n6-SAL	6.51 (3.4–24.7)**	5 (3–9)	1.11 (0.6–4.2)**	7.81 (5.6–13.8)
n6-POL	1.4 (0.3–2.8)**	2 (0.5–4)	0.24 (0.05–0.5)**	3.28 (1–5.4)
n3-SAL	7.71 (3.3–11.3)	6 (3.5–6.8)	1.3 (0.5–1.9)	9.94 (6.8–12.9)
n3-POL	12.9 (1.6–49.6)	4 (1.3–10.3)	2.18 (0.3–8.4)	5.92 (1.9–15.7)

Abbreviations: EPM, elevated plus maze; MIA, maternal immune activation; n6-POL, prenatal PolyI:C-exposed offspring treated with *n-6* PUFA; n3-SAL, prenatal saline-exposed offspring treated with *n-3* PUFA; n6-SAL, prenatal saline-exposed offspring treated with n6-polyunsaturated fatty acids (*n-6* PUFA) control diet; PUFA, polyunsaturated fatty acids; Sal, saline.

**P⩽0.01.

All values are median (25–75 percentiles).

**Table 3:** Weight and body mass measurements in adulthood in MIA mice with or without *n-3* PUFA diet interventions in peri-adolescence

*Groups*	*Weight (g)*	*Fat (g)*	*% Fat*	*Lean (g)*	*% Lean*	*Fluid (g)*	*% Fluid*
n6-SAL	38.6±1.77**	11.3±0.78	29.2±1.2	19.3±0.87**	50±1.41	3.9±0.24**	10.1±0.46
n6-POL	38.3±2.22**	11.0±0.96	28.7±1.61	19.6±1.29**	51.1±1.89	4.0±0.27**	10.4±0.53
n3-SAL	41.8±1.66**	12.1±1.05	28.9±1.86	21.4±0.87**	51.2±1.82	4.3±0.16**	10.4±0.45
n3-POL	38.6±2.31**	11.3±0.86	29.3±0.68	19.9±1.03**	51.6±0.89	4.0±0.26**	10.3±0.54

Abbreviations: MIA, maternal immune activation; n6-POL, prenatal PolyI:C-exposed offspring treated with *n-6* PUFA; n3-SAL, prenatal saline-exposed offspring treated with *n-3* PUFA; n6-SAL, prenatal saline-exposed offspring treated with n6-polyunsaturated fatty acids (*n-6* PUFA) control diet; PUFA, polyunsaturated fatty acids; Sal, saline.

**P<0.01

% Fat (fat/weight), % lean (lean/weight), % fluid (fluid/weight).

All values are mean±s.d.
