# Supplementary material for: Dietary supplementation with n-3 fatty acids from weaning limits brain biochemistry and behavioural changes elicited by prenatal exposure to maternal inflammation in the mouse model
Source: Transl Psychiatry. 2015 Sep 22;5(9):e641–. doi: 10.1038/tp.2015.126 (PMC5068805; doi:10.1038/tp.2015.126)
Supplement: Supplementary Table 1 [file tp2015126x1.pdf]

Supplementary Table 1. Summary of the MRS metabolites in adulthood in MIA mice with or without *n-3* PUFA interventions in peri-adolescence

Groups	MRS Metabolites				
	Cho/Cr	Glx/Cr	Gly/Cr	Lac/Cr	Tau/Cr
n6-SAL	0.18 ± 0.04	1.21 ± 0.09	0.87 ± 0.15	0.42 ± 0.08	1.92 ± 0.05
n6-POL	0.20 ± 0.03	1.11 ± 0.07	0.53 ± 0.10	0.41 ± 0.07	1.71 ± 0.14
n3-SAL	0.13 ± 0.02	1.09 ± 0.11	0.59 ± 0.07	0.35 ± 0.05	1.78 ± 0.10
n3-POL	0.13 ± 0.02	1.19 ± 0.07	0.66 ± 0.10	0.26 ± 0.04	1.90 ± 0.07

All values are means ± SEM. Groups: n6-SAL: Prenatal saline-exposed offspring treated with n6-polyunsaturated fatty acids (n-6 PUFA) control diet; n6-POL: Prenatal PolyI:C-exposed offspring treated with n-6 PUFA; n3-SAL: Prenatal saline-exposed offspring treated with n-3 PUFA; n3-POL: Prenatal PolyI:C-exposed offspring treated with n-3 PUFA. Cho: choline; Cr: creatine; GLX: glutamate + glutamine; Gly: glycine; Lac: lactatae; Tau: taurine.
