# Supplementary material for: Dietary supplementation with n-3 fatty acids from weaning limits brain biochemistry and behavioural changes elicited by prenatal exposure to maternal inflammation in the mouse model
Source: Transl Psychiatry. 2015 Sep 22;5(9):e641–. doi: 10.1038/tp.2015.126 (PMC5068805; doi:10.1038/tp.2015.126)
Supplement: Supplementary Figures [file tp2015126x2.docx]

**Supplementary Figure 1**

**B.**

**A.**

**C.**

**Supplementary Figure 1A.** Mean startle reactivity across 10 pulse-alone trails presented in the middle of the session at 100dB pulse (P100), 110dB pulse (P110) and 120dB pulse (P120). **B.** Mean prepulse reactivity across 10 pre-pulse-alone trails presented in the middle of the session at prepulse 71dB,77dB and 83dB.

Groups: n6-SAL: Prenatal saline-exposed offspring treated with n6-polyunsaturated fatty acids (n-6 PUFA) control diet; n6-POL: Prenatal PolyI:C-exposed offspring treated with n-6 PUFA; n3-SAL: Prenatal saline-exposed offspring treated with n-3 PUFA; n3-POL: Prenatal PolyI:C-exposed offspring treated with n-3 PUFA.

**Supplementary Figure 2**

**Supplementary Figure 2. Locomotor response to amphetamine challenge in the open field test.**

Total activity for 30 min after saline administration vs. locomotor response to amphetamine (Amph) (2.5 mg/kg) for 90 min (30 min/block, 3 blocks. Block 1 means the total distance moved the first 30min -, Block 2 means the second 30min -, Block 3 means the third 30min -, after Amph injection) expressed in the different groups. All values are means ± SEM.

Groups: n6-SAL: Prenatal saline-exposed offspring treated with n6-polyunsaturated fatty acids (n-6 PUFA) control diet; n6-POL: Prenatal PolyI:C-exposed offspring treated with n-6 PUFA; n3-SAL: Prenatal saline-exposed offspring treated with n-3 PUFA; n3-POL: Prenatal PolyI:C-exposed offspring treated with n-3 PUFA.

**Supplementary Figure 3**


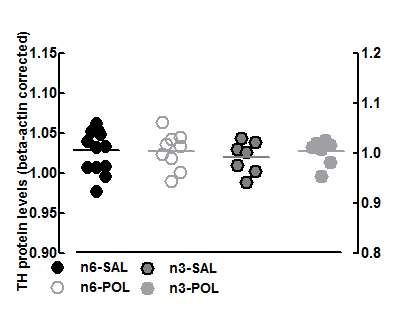

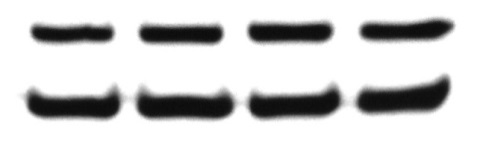


**TH**

**β-actin**

**n6-SAL**

**n6-POL**

**n3-SAL**

**n3-POL**

**NAc**


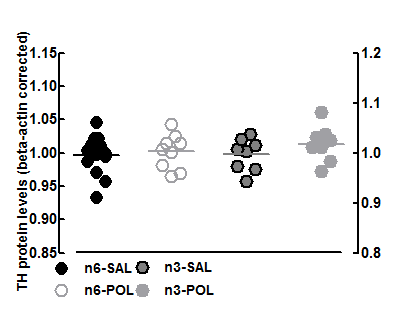

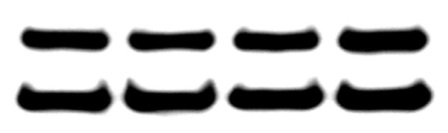


**TH**

**β-actin**

60kDa

42kDa

**n6-SAL**

**n6-POL**

**n3-POL**

**n3-SAL**

**CP**


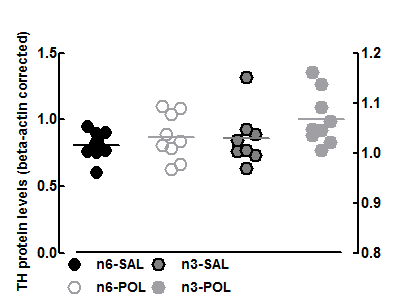

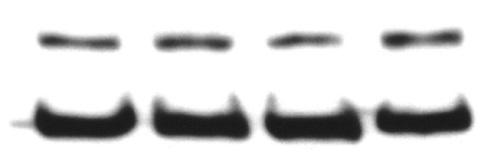


**TH**

**β-actin**

**n6-SAL**

**n6-POL**

**n3-SAL**

**n3-POL**

**mPFC**

**A** **B** **C**


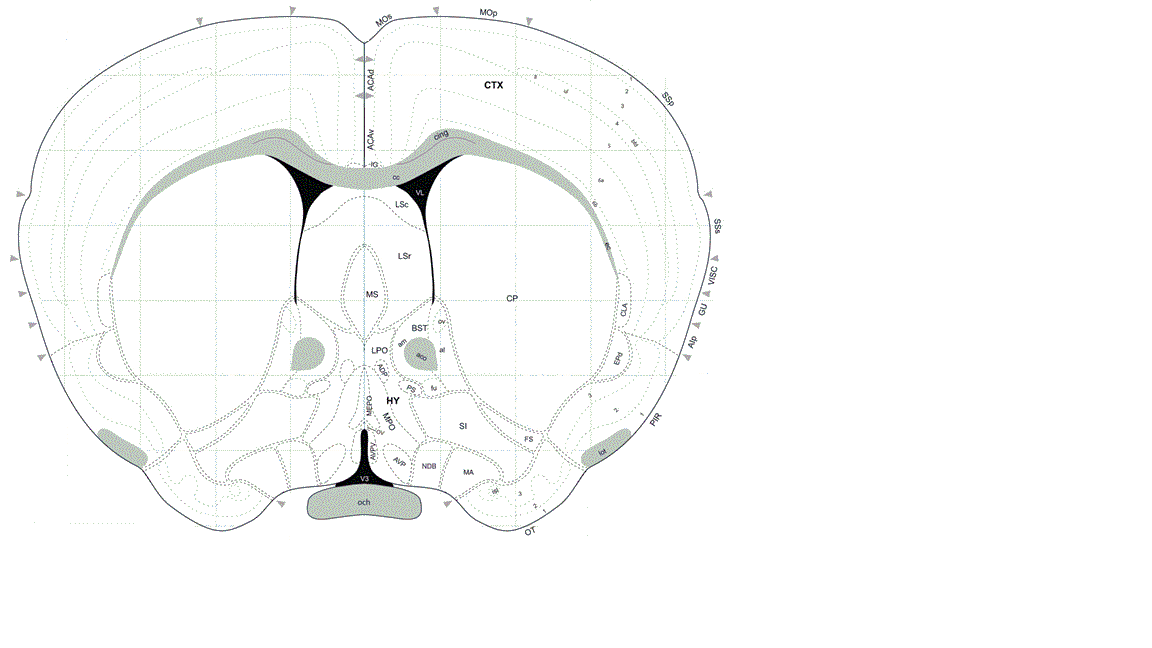

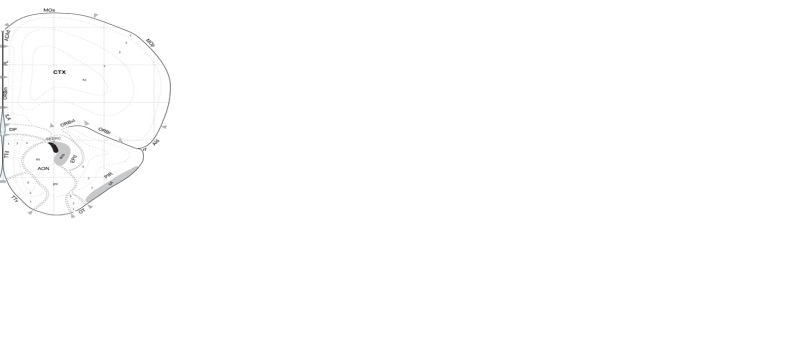


**Supplementary Figure 3. Protein levels of TH in A. caudate putamen (CP), B. nucleus accumbens NAc) and C. medial prefrontal cortex (mPFC) in adult offspring.** β-actin is shown as control for comparison. All values are means ± SEM.

Groups: n6-SAL: Prenatal saline-exposed offspring treated with n6-polyunsaturated fatty acids (n-6 PUFA) control diet; n6-POL: Prenatal PolyI:C-exposed offspring treated with n-6 PUFA; n3-SAL: Prenatal saline-exposed offspring treated with n-3 PUFA; n3-POL: Prenatal PolyI:C-exposed offspring treated with n-3 PUFA.
